# Sex-specific outcome disparities in very old patients admitted to intensive care medicine: a propensity matched analysis

**DOI:** 10.1038/s41598-020-74910-3

**Published:** 2020-10-29

**Authors:** Bernhard Wernly, Raphael Romano Bruno, Malte Kelm, Ariane Boumendil, Alessandro Morandi, Finn H. Andersen, Antonio Artigas, Stefano Finazzi, Maurizio Cecconi, Steffen Christensen, Loredana Faraldi, Michael Lichtenauer, Johanna M. Muessig, Brian Marsh, Rui Moreno, Sandra Oeyen, Christina Agvald Öhman, Bernado Bollen Pinto, Ivo W. Soliman, Wojciech Szczeklik, David Niederseer, Andreas Valentin, Ximena Watson, Susannah Leaver, Carole Boulanger, Sten Walther, Joerg C. Schefold, Michael Joannidis, Yuriy Nalapko, Muhammed Elhadi, Jesper Fjølner, Tilemachos Zafeiridis, Dylan W. De Lange, Bertrand Guidet, Hans Flaatten, Christian Jung

**Affiliations:** 1 Department of Cardiology, Paracelsus Medical University, Salzburg, Austria; 2 Division of Cardiology, Department of Medicine, Karolinska Institutet, Karolinska University Hospital, Stockholm, Sweden; 3 Department of Cardiology, Pulmonology and Angiology, University Hospital, Moorenstraße 5, 40225 Duesseldorf, Germany; 4 Service de Réanimation Médicale, Publique-Hôpital de Paris, Hôpital Saint-Antoine, 75012 Paris, France; 5 Department of Rehabilitation, Hospital Ancelle Di Cremona, Cremona, Italy; 6 Geriatric Research Group, Brescia, Italy; 7 Department of Anaesthesia and Intensive Care, Ålesund Hospital, Ålesund, Norway; 8 Department of Circulation and Medical Imaging, NTNU, Trondheim, Norway; 9 Department of Intensive Care Medecine, CIBER Enfermedades Respiratorias, Corporacion Sanitaria Universitaria Parc Tauli, Autonomous University of Barcelona, Sabadell, Spain; 10 Department of Intensive Care Medecine, University Hospitals Sagrado Corazón and General de Catalunya, Quirón Salud, Barcelona-Sant Cugat, Spain; 11 Dipartimento Di Epidemiologia Clinica, IRCCS Istituto Di Ricerche Farmacologiche “Mario Negri”, Ranica, BG Italy; 12 Department of Anaesthesia IRCCS, Instituto Clínico Humanitas, Humanitas University, Milan, Italy; 13 Department of Anaesthesia and Intensive Care Medicine, Aarhus University Hospital, Aarhus, Denmark; 14 Grande Ospedale Metropolitano Niguarda, Milan, Italy; 15 Misericordiae University Hospital, Dublin, Ireland; 16 Centro Hospitalar Universitário de Lisboa Central, Nova Médical School, Faculdade de Ciências Médicas de Lisboa, Unidade de Cuidados Intensivos Neurocríticos E Trauma, Hospital de São José, Lisbon, Portugal; 17 Department of Intensive Care 1K12IC, Ghent University Hospital, Ghent, Belgium; 18 Karolinska University Hospital, Stockholm, Sweden; 19 Geneva University Hospitals, Geneva, Switzerland; 20 Department of Intensive Care Medicine, University Medical Center, University Utrecht, Utrecht, The Netherlands; 21 Intensive Care and Perioperative Medicine Division, Jagiellonian University Medical College, Kraków, Poland; 22 Department of Cardiology, University Heart Center Zurich, University Hospital Zurich, University of Zurich, Zurich, Switzerland; 23 Kardinal Schwarzenberg Hospital, Schwarzach, Austria; 24 St George’s University Hospital, London, UK; 25 Research Lead Critical Care Directorate St George’s Hospital, London, UK; 26 NAHP Section ESICM,Intensive Care Unit, Royal Devon & Exeter NHS Foundation Trust, Exeter, UK; 27 Linkoping University Hospital, Linkoping, Sweden; 28 Inselspital, Bern University Hospital, Bern, Switzerland; 29 Division of Intensive Care and Emergency Medicine, Department of Internal Medicine, Medical University Innsbruck, Innsbruck, Austria; 30 European Wellness International, ICU, Luhansk, Ukraine; 31 Alkhums Hospital, ICU, Tripoli, Libya; 32 Department of Intensive Care, Aarhus University Hospital, Aarhus, Denmark; 33 Intensive Care Unit General Hospital of Larissa Tsakalof Larissa, Larissa, Greece; 34 Service de Réanimation, INSERM, Institut Pierre Louis d’Epidémiologie et de Santé Publique, AP-HP, Sorbonne Universités, 75013 Paris, France; 35 UMR_S 1136, Institut Pierre Louis D’Epidémiologie Et de Santé Publique, INSERM, 75013 Paris, France; 36 Department of Clinical Medecine, University of Bergen, Bergen, Norway; 37 Department of Anaestesia and Intensive Care, Haukeland University Hospital, Bergen, Norway

**Keywords:** Health care, Medical research

## Abstract

Female and male very elderly intensive patients (VIPs) might differ in characteristics and outcomes. We aimed to compare female versus male VIPs in a large, multinational collective of VIPs with regards to outcome and predictors of mortality. In total, 7555 patients were included in this analysis, 3973 (53%) male and 3582 (47%) female patients. The primary endpoint was 30-day-mortality. Baseline characteristics, data on management and geriatric scores including frailty assessed by Clinical Frailty Scale (CFS) were documented. Two propensity scores (for being male) were obtained for consecutive matching, score 1 for baseline characteristics and score 2 for baseline characteristics and ICU management. Male VIPs were younger (83 ± 5 vs. 84 ± 5; *p* < 0.001), less often frail (CFS > 4; 38% versus 49%; *p* < 0.001) but evidenced higher SOFA (7 ± 6 versus 6 ± 6 points; *p* < 0.001) scores. After propensity score matching, no differences in baseline characteristics could be observed. In the paired analysis, the mortality in male VIPs was higher (mean difference 3.34% 95%CI 0.92–5.76%; *p* = 0.007) compared to females. In both multivariable logistic regression models correcting for propensity score 1 (aOR 1.15 95%CI 1.03–1.27; *p* = 0.007) and propensity score 2 (aOR 1.15 95%CI 1.04–1.27; *p* = 0.007) male sex was independently associated with higher odds for 30-day-mortality. Of note, male gender was not associated with ICU mortality (OR 1.08 95%CI 0.98–1.19; *p* = 0.14). Outcomes of elderly intensive care patients evidenced independent sex differences. Male sex was associated with adverse 30-day-mortality but not ICU-mortality. Further research to identify potential sex-specific risk factors after ICU discharge is warranted.

**Trial registration**: NCT03134807 and NCT03370692; Registered on May 1, 2017 https://clinicaltrials.gov/ct2/show/NCT03370692.

## Introduction

Patients 80 years of age and older, who are admitted to the intensive care unit (ICU) consume a large proportion of health care resources and yet continue to suffer from high mortality^1–3^. Detailed knowledge of these very elderly intensive patients (VIPs) could help to perform better risk stratification and ultimately guide clinicians in whom to admit or whom not to admit to the ICU. The Clinical Frailty Scale (CFS), evaluating frailty through a simple clinical assessment, has been shown to adequately risk-stratify such elderly patients^4,5^.

For several medical conditions, including acute myocardial infarction, gender outcome disparities have been reported^6^. However, some studies investigated gender differences in ICU patients, and have found distinct differences^7,8^. Male and female intensive care patients differ with regards to baseline characteristics, risk distribution and admission diagnoses and these differences may influence outcomes^9,10^. Male sex was linked to adverse outcomes in a sub-set of VIPs with sepsis^10,11^. On the other hand, female sex was reported to be independently associated with the decision to withdraw or withhold intensive care^12^. Recently, the FROG-ICU evaluated survival in critically ill patients and reported a trend towards higher survival in elderly women compared to male patients^13^.

We, therefore, aimed to compare male versus female VIPs with regards to the distribution of risk factors, potential differences in management, and outcome as well as predictors of mortality with special emphasis on frailty. The main goal with this study using data from two recent large, multinational studies of VIPs was to compare male and female patients with regards to crude unadjusted und adjusted baseline characteristics and outcomes^4,14,15^.

## Methods

### Study subjects

VIP1 and VIP2 were prospective, multicenter studies, registered on ClinicalTrials.gov (ID: NTC03134807, NCT03370692). Both studies included very old intensive care patients (VIPs), defined as patients admitted to an ICU and being aged 80 years or older. These patients have been analyzed in other contexts and methods and results have been published previously^4,5,16^. In summary, for VIP1, each participating ICU could include either consecutive patients during three months or the first 20 consecutive patients fulfilling the inclusion criteria (all patients 80 of age or older). Data were collected between October 2016 and February 2017. For VIP2, VIPs were included from May 2018 to May 2019. All methods were carried out in accordance with relevant guidelines and regulations. All experimental protocols were approved by the local institutional and/or licensing committees. Informed consent was obtained from all subjects if not omitted by the ethics vote.

In this post-hoc analysis of these two prospective trials, all patients admitted acutely (non-electively) with complete data on age, gender, clinical frailty score (CFS) frailty score and sequential organ failure assessment (SOFA) score and 30-day-mortality were included (Supplemental Fig. 1). Elective patients from VIP1 were specifically excluded as they significantly differ from acutely admitted patients in risk distribution and outcomes as previously shown^17^. The primary endpoint of this study was 30-day-mortality. Frailty was assessed by CFS and the respective visual and simple description which were used with permission^18–20^. For the patients of the VIP2 trial Katz activities of daily living (Katz ADL) with ADL score ≤ 4 defining disability and Short form of Informant Questionnaire on Cognitive Decline in the Elderly (IQCODE), with IQCODE ≥ 3.5 defining cognitive decline were assessed^18–20^.

**Figure 1:**
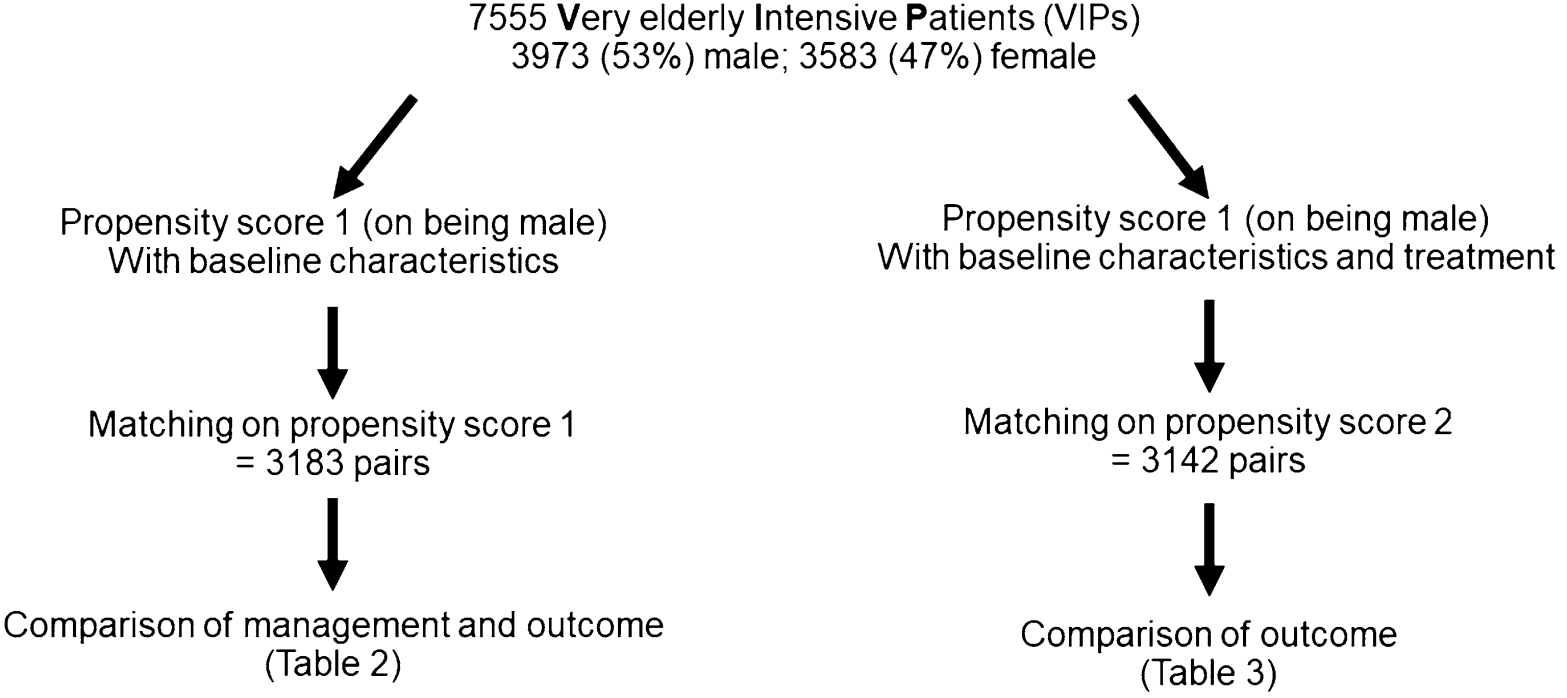
Flow chart of the propensity-score matching process.

### Statistical analysis

Continuous data points are expressed as mean ± standard deviation (SD) or median ± interquartile range depending on distribution. Differences between independent groups were calculated using student’s T-test or Mann Whitney U-test accordingly. Categorical data are expressed as numbers (percentage). Chi-square test was applied to calculate differences between groups and McNemar’s test for paired survival data.

Two propensity scores for being male were calculated (Fig. 1). Propensity score 1 included age (per year), CFS score (per point), SOFA score (per point), location (Western Europe, Eastern Europe, Non-European) and admission diagnosis (respiratory failure, circulatory failure, combined respiratory and circulatory failure, sepsis, multi-trauma with and without head injury, isolated head injury, intoxication, cerebral injury without trauma, emergency surgery, other). Propensity score 2 included all items of propensity score 1 plus the non-baseline variables use of vasoactive drugs, of renal replacement therapy, of intubation, of non-invasive ventilation (NIV) as well as the decision to limit life-sustaining treatment (withdrawal and/or withholding). Two matched cohorts, matching males 1:1 to females, for (1) propensity score 1 and (2) propensity score 2 were obtained using “nearest neighbor” matching, the maximum allowed distance was a Δ in propensity score 1 or 2 of 0.001. The matching significantly reduced differences in baseline characteristics and management.

Sensitivity analysis, analyzing only patients without treatment restrictions and European patients, was performed. Univariable and multivariable logistic regression analysis was performed to assess associations with treatment withdrawal and mortality. Odds ratios (OR) and adjusted odds ratios (aOR) with respective 95% confidence intervals (CI) were calculated. Two multivariable logistic regression models were built for the total cohort, (1) using propensity score 1 and (2) using propensity score 2 as covariable. For the sub-group analysis assessing associations of parameters with 30-day-mortality in male and female patients, variables with a *p* value < 0.10 in the univariable analysis were included in the multivariable model, then a backward elimination was performed, the elimination criterion was 0.10. All tests were two-sided, and a *p* value of < 0.05 was considered statistically significant. SPSS version 23.0 (IBM, USA) and MedCalc Statistical Software version 19.1.3 (MedCalc Software bv, Ostend, Belgium; https://www.medcalc.org; 2019) were used for all statistical analyses.

### Ethics approval and consent to participate

A study protocol was provided to participating centers. Every participating center obtained ethics approval according to local legislation. A copy of the ethics approval was sent to the study coordinator before start of the study.

### Consent for publication

Written informed consent was obtained of all included subjects, except for patients from VIP2 of sites where study inclusion was explicitly granted without written informed consent.

## Results

### Study population

In total, 7555 patients were included in this analysis, 3973 (53%) male and 3582 (47%) female patients. Admission diagnoses and baseline characteristics are presented in Table 1. Male patients were younger compared to female patients, with fewer male patients being over 90 years of age (6% vs. 8%; *p* < 0.001). Male patients were less often frail (CFS > 4; 38% vs. 49%; *p* < 0.001) and less often suffered from disability (ADL ≤ 4; 25% vs. 31%; *p* < 0.001), and cognitive decline (IQCODE ≥ 3.5; 29% vs. 36%; *p* < 0.001).

**Table 1:** Baseline characteristics in the total cohort, male versus female VIPs.

	Male	Female	*p* value
	n = 3973	n = 3582	
**Age**			
Median (± IQR)	83 (5)	84 (5)	< 0.001
Age > 90 n (%)	227 (5.7%)	288 (8%)	< 0.001
**Frailty score—CFS**			
Median (± IQR)	4 (2)	4 (3)	< 0.001
Frailty (CFS > 4) n (%)	1519 (38%)	1754 (49%)	< 0.001
**ADL**			
Median (± IQR)	6 (1)	6 (2)	< 0.001
Disablitiy (ADL ≤ 4)	446 (25%)	489 (31%)	< 0.001
**IQCODE**			
Median (± IQR)	3.2 (0.6)	3.3 (0.8)	0.001
Cognitive decline (IQCODE ≥ 3.5)	455 (29%)	486 (36%)	< 0.001
median (± IQR)	7 (6)	6 (6)	< 0.001
**ICU length of stay (hours)**			
median (± IQR)	89 (154)	72 (131)	< 0.001
Treatment withdraw and/or withold (%)	1235 (35)	1342 (34)	0.53
NIV n (%)	933 (25%)	873 (24%)	0.54
Intubation n (%)	2108 (53%)	1728 (48%)	< 0.001
Renal replacement therapy n (%)	530 (13%)	296 (8%)	< 0.001
Vasoactive drugs n (%)	2397 (60%)	2038 (57%)	0.003
**Admission diagnosis**			
Respiratory failure	928 (23%)	889 (25%)	< 0.001
Circulatory failure	577 (15%)	490 (14%)	
Combined circulatory and respiratory failure	493 (12%)	395 (11%)	
Sepsis	555 (14%)	451 (13%)	
Multitrauma w/o head injury	82 (2%)	58 (2%)	
Trauma with head injury	74 (2%)	57 (2%)	
Head injury	100 (3%)	83 (2%)	
Intoxication	12 (< 1%)	24 (1%)	
Cerebral injury (non-traumatic)	231 (6%)	248 (7%)	
Emergency surgery	442 (6%)	464 (13%)	
Other	479 (12%)	423 (12%)	

*CFS* Clinical Frailty Scale, *SOFA* Sequential Organ Failure Assessment, *ADL* Activity of Daily Life measured with the Katz Index, *IQCODE* Informant Questionnaire on COgnitive Decline in the Elderly, *ICU* intensive care unit, *NIV* non-invasive ventilation, *SD* standard deviation.

Rates of non-invasive ventilation usage (NIV; 25% vs. 24%; *p* = 0.29) did not differ between male and female patients. Rates of intubation (53% vs. 48%; *p* < 0.001), renal replacement therapy (13% vs. 8%; *p* < 0.001) and vasoactive drugs (60% vs. 57%; *p* = 0.003) were higher in male patients compared to females.

Organ failures as assessed by SOFA score was higher in male patients (7 ± 6 vs. 6 ± 6 points; *p* < 0.001) and the length of ICU stay was longer (89 ± 154 vs. 72 ± 131 h; *p* < 0.001).

The rates of life-sustainment limitation were similar (35% vs. 34%; *p* = 0.53). In multivariable logistic regression model, after correction for propensity score 1, male gender was not independently associated with any treatment limitation (aOR 0.92 95%CI 0.83–1.03; *p* = 0.14).

### Survival analysis in the total cohort

In univariable analysis in the unbalanced total cohort, 30-day-mortality was higher (43% vs. 39%; OR 1.18 95%CI 1.08–1.30; *p* < 0.001) in male patients compared to female patients. In multivariable logistic regression models correcting for propensity score 1 (aOR 1.15 95%CI 1.03–1.27; *p* = 0.007) as well as propensity score 2 (aOR 1.15 95%CI 1.04–1.27; *p* = 0.007) male gender was independently associated with higher odds for 30-day-mortality. Also, after adjustment for propensity score 2 and length of ICU stay, male sex (aOR 1.13 95%CI 1.03–1.24; *p* = 0.01) remained independently associated with higher odds for 30-day-mortality.

In sensitivity analysis excluding patients with treatment limitation, after correction for propensity score 1 male gender was independently associated with mortality (aOR 1.19 95%CI 1.04–1.38; *p* = 0.02) and remained so in trend after correction for propensity score 2 (aOR 1.15 95%CI 0.996–1.326; *p* = 0.056). In sensitivity analysis excluding non-European countries, male gender was independently associated with higher rates of 30-day-mortality after correction for propensity score 1 (aOR 1.14 95%CI 1.03–1.26; *p* = 0.01) and propensity score 2 (aOR 1.14 95%CI 1.03–1.27; *p* = 0.01). Of note, male gender was not associated with ICU mortality (OR 1.08 95%CI 0.98–1.19; *p* = 0.14).

### Matched-cohort 1

Baseline characteristics of the matched-cohort 1 (matched on propensity score 1, which included only baseline variables, see Fig. 1) are given in Table 2. Risk parameters were evenly distributed between male and female patients, but rates of renal replacement therapy were higher (13% vs. 9%; *p* < 0.001) in males as were lengths of ICU stay.

**Table 2:** Baseline characteristics in the matched cohort 1, male versus female VIPs.

	Male	Female	*p* value
	n = 3183	n = 3183	
**Age**			
Median (± IQR)	84 (6)	84 (6)	0.91
Age > 90 n (%)	207 (7%)	195 (6%)	0.57
**Frailty score—CFS**			
Median (± IQR)	4 (3)	4 (3)	0.94
Frailty (CFS > 4) n (%)	1379 (43%)	1409 (44%)	0.46
**ADL**			
Median (± IQR)	6 (2)	6 (2)	0.40
Disablitiy (ADL ≤ 4)	400 (28%)	375 (27%)	0.56
**IQCODE**			
Median (± IQR)	3.3 (0.7)	3.3 (0.7)	0.92
Cognitive decline (IQCODE ≥ 3.5)	404 (32%)	404 (33%)	0.49
**SOFA score**			
Median (± IQR)	7 (6)	6 (6)	0.19
**ICU length of stay (hours)**			
Median (± IQR)	86 (151)	72 (132)	< 0.001
Treatment withdraw and/or withold (%)	1054 (33%)	1111 (35%)	0.15
NIV n (%)	789 (25%)	784 (25%)	0.88
Intubation n (%)	1623 (51%)	1559 (49%)	0.10
Renal replacement therapy n (%)	397 (13%)	277 (9%)	< 0.001
Vasoactive drugs n (%)	1846 (58%)	1850 (58%)	0.92
**Admission diagnosis**			
Respiratory failure	783 (25%)	786 (25%)	0.99
Circulatory failure	445 (14%)	449 (14%)	
Combined circulatory and respiratory failure	353 (11%)	356 (11%)	
Sepsis	413 (13%)	410 (13%)	
Multitrauma w/o head injury	63 (2%)	55 (2%)	
Trauma with head injury	52 (2%)	55 (2%)	
Head Injury	78 (3%)	74 (2%)	
Intoxication	8 (< 1%)	14 (< 1%)	
Cerebral injury (non-traumatic)	203 (6%)	199 (6%)	
Emergency surgery	393 (12%)	391 (12%)	
Other	392 (12%)	391 (12%)	

*CFS* Clinical Frailty Scale, *SOFA* Sequential Organ Failure Assessment, *ADL* Activity of Daily Life measured with the Katz Index, *IQCODE* Informant Questionnaire on COgnitive Decline in the Elderly, *ICU* intensive care unit, *NIV* non-invasive ventilation, *SD* standard deviation.

In the paired analysis, the mortality in male VIPs was higher (mean difference 3.33% 95%CI 0.92–5.74%; *p* = 0.007) compared to females. In univariable logistic regression, male gender was associated with higher odds for 30-day-mortality (42% vs. 38%; OR 1.15 95%CI 1.04–1.27; *p* = 0.007). Again, male gender was not (OR 1.02 95%CI 0.92–1.14; *p* = 0.69) associated with intra-ICU mortality in this matched cohort.

### Matched-cohort 2

Table 3 shows baseline characteristics of matched-cohort 2 (matched on propensity score 2, which includes baseline variables and information on organ support as well as treatment limitations, see Fig. 1). Again, male patients evidenced longer ICU stays (*p* < 0.001).

**Table 3:** Baseline characteristics in the matched cohort 2, male versus female VIPs.

	Male	Female	*p* value
	n = 3142	n = 3142	
**Age**			
Mean (± SD)	84 (5)	84 (6)	0.61
Age > 90 n (%)	213 (7%)	207 (7%)	0.80
**Frailty score—CFS**			
Mean (± SD)	4 (3)	4 (3)	0.60
Frailty (CFS > 4) n (%)	1355 (43%)	1406 (45%)	0.20
**ADL**			
Mean (± SD)	6 (2)	6 (2)	0.40
Disablitiy (ADL ≤ 4)	390 (27%)	366 (27%)	0.73
**IQCODE**			
Mean (± SD)	3.2 (0.7)	3.3 (0.7)	0.41
Cognitive decline (IQCODE ≥ 3.5)	392 (32%)	390 (33%)	0.38
**SOFA score**			
Mean (± SD)	7 (6)	6 (6)	0.48
**ICU length of stay (hours)**			
Mean (± SD)	78 (136)	72 (133)	0.007
Treatment withdraw and/or withold (%)	1077 (34%)	1080 (34%)	0.96
NIV n (%)	779 (25%)	792 (25%)	0.73
Intubation n (%)	1566 (50%)	1548 (49%)	0.67
Renal replacement therapy n (%)	287 (9%)	285 (9%)	0.93
Vasoactive drugs n (%)	1825 (58%)	1819 (58%)	0.90
**Admission diagnosis**			
Respiratory failure	766 (24%)	781 (25%)	
Circulatory failure	453 (14%)	441 (14%)	
Combined circulatory and respiratory failure	361 (12%)	362 (12%)	
Sepsis	406 (13%)	418 (13%)	
Multitrauma w/o head injury	53 (2%)	57 (2%)	
Trauma with head injury	51 (2%)	50 (2%)	
Head injury	79 (3%)	80 (3%)	
Intoxication	7 (< 1%)	7 (< 1%)	
Cerebral injury (non-traumatic)	200 (6%)	195 (6%)	
Emergency surgery	397 (13%)	375 (12%)	
Other	369 (12%)	376 (12%)	
Other	369 (12%)	376 (12%)	

*CFS* Clinical Frailty Scale, *SOFA* sequential organ failure assessment, *ADL* Activity of Daily Life measured with the Katz Index, *IQCODE* Informant Questionnaire on COgnitive Decline in the Elderly, *ICU* intensive care unit, *NIV* Non-invasive ventilation, *SD* standard deviation.

Again, in the paired analysis, the mortality in male VIPs was higher (mean difference 3.34% 95%CI 0.92–5.76%; *p* = 0.007) compared to females. In univariable logistic regression, male gender was associated with higher odds for 30-day-mortality (42% vs. 39%; aOR 1.15 95%CI 1.04–1.27; *p* = 0.007). Again, gender was not associated with ICU mortality (OR 1.02 95%CI 0.92–1.14; *p* = 0.67) in this matched cohort.

### Sub-group analysis of female and male patients

The presence of frailty (CFS > 4) was associated with increased 30-day-mortality in male patients (OR 1.73 95%CI 1.52–1.97; *p* < 0.001) and remained so in multivariable logistic regression (Table 4a).

**Table 4:** Associations of relevant factors with 30-day mortality in (a) male patients and (b) female patients.

	Univariable			Multivariable		
	OR	95%CI	*p* value	aOR	95%CI	*p* value
**a**						
*Male patients*						
Age (per year)	1.04	1.02–1.05	< 0.001	1.03	1.01–1.05	0.02
SOFA (per point)	1.18	1.16–1.20	< 0.001	1.11	1.08–1.13	< 0.001
Frailty (per CFS point)	1.21	1.16–1.25	< 0.001	1.13	1.09–1.18	< 0.001
Vasoactive drug (yes vs. no)	2.44	2.13–2.79	< 0.001	0.98	0.81–1.19	0.87
Intubation (yes vs. no)	2.75	2.41–3.14	< 0.001	2.34	1.97–2.78	< 0.001
Renal replacement therapy (yes vs. no)	2.05	1.71–2.47	< 0.001	1.58	1.27–1.98	0.001
Treatment withdrawal or withholding (yes vs. no)	9.02	7.74–10.51	< 0.001	8.99	7.62–10.61	< 0.001
**b**						
*Female patients*						
Age (per year)	1.02	1.002–1.038	0.03	1.02	0.99–1.04	0.17
SOFA (per point)	1.22	1.19–1.14	< 0.001	1.14	1.11–1.17	< 0.001
Frailty (per CFS point)	1.22	1.17–1.27	< 0.001	1.16	1.10–1.21	< 0.001
Vasoactive drug (yes/no)	3.09	2.67–3.57	< 0.001	1.27	1.03–1.55	0.02
Intubation (yes/no)	3.63	3.15–4.18	< 0.001	2.53	2.08–3.07	< 0.001
Renal replacement therapy (yes/no)	3.52	2.73–4.52	< 0.001	3.34	1.74–3.15	< 0.001
Treatment withdrawal or withholding (yes/no)	6.95	5.96–8.10	< 0.001	8.15	6.83–9.72	< 0.001

*OR* odds ratio, *aOR* adjusted OR, *SOFA* Sequential Organ Failure Assessment, *CFS* Clinical Frailty Scale.

In female patients frailty (CFS > 4) was associated with 30-day mortality in univariable analysis (OR 1.65 95%CI 1.44–1.89; *p* < 0.001) and remained so after correction in multivariable logistic regression (Table 4b).

Furthermore, one-point increases of CFS, as well as SOFA, were independently associated with increased odds for 30-day-mortality in multivariable logistic regression in male (Table 4a) as well as in female (Table 4b) VIPs.

## Discussion

In this post-hoc analysis of a large group of VIPs included in two international ICU prospective studies, differences in the distribution of baseline characteristics and risk factors between male and female patients could be found. Further, male sex was associated with increased 30-day-mortality in VIPs and remained so after propensity-score adjustments for both baseline characteristics alone and baseline characteristics as well as in-ICU variables. However, sex was not associated with ICU-mortality, neither in the total cohort nor in the adjusted matched cohorts.

Frailty assessed by CFS was independently associated with 30-day-mortality both in male and female patients after adjustment for baseline risk factors. Therefore, CFS could safely be integrated in guiding pre-ICU triage as well as intra-ICU triage both in male and female patients.

Male and female patients differed with regards to baseline characteristics, management, and outcomes. Male VIPs in this cohort were younger and evidenced lower rates of frailty, disability and cognitive impairment. On the other hand, male patients were clinically sicker as expressed by higher SOFA scores. Consequently, unadjusted 30-day-mortality was higher in male compared to female VIPs. After adjustment for baseline characteristics, except for rates of renal replacement therapy, there were no differences in the management of organ support between male and female patients. Of note, there are recent data indicating higher susceptibility of kidney to injury in male epithelial cells as compared to female^21^. Importantly, the rates of treatment limitation did not differ between male and female VIPs, nor after adjustment in multivariable analysis in the total cohort neither in the propensity-matched cohorts.

However, after matching and adjustment for both baseline characteristics alone as well as baseline characteristics plus ICU management, male gender was still independently associated with increased 30-day-mortality in this analysis. Further, male gender remained independently associated with increased 30-day-mortality in a sensitivity analysis excluding patients with treatment limitations. Importantly, these results confirm observed trends in a recent sub-study of the FROG-ICU study: Hollinger et al. reported increased survival rates in moderately elderly women compared to men, whereas in the overall cohort consisting of more than 2000 critically ill patients no sex-related differences in outcomes could be found^13^. These findings, relating male gender to adverse outcomes, are consistent with previous studies reporting adverse outcomes in male septic VIPs^10^. On the other hand, this trend in gender difference was not observed for illness-adjusted mortality in a large Austrian cohort study on 25,998 patients without age-restriction^22^. Therefore, the observed sex differences in mortality could be age-dependent.

Several factors could contribute to this finding. Certainly, bias and lack of data need to be considered, although extensive adjustment for baseline characteristics as well as treatment management was performed using propensity scores. However, importantly, only adjustment to available and known covariables is possible. First, this study lacks extensive data on comorbidities, which probably influence management and outcome^23^. However, adjustments for frailty, which is associated with the amount and extent of comorbidities, were performed. Second, further sensitivity analysis and adjustment on both macro- microcirculatory parameters could have improved our understanding of this cohort as men have a shorter life expectancy and die at a younger age: Their bodies are more worn at a same age which could be underestimated in categorical datasets, like SOFA and CFS: continuous data (like biomarkers) could pick up this difference^24^. Especially biomarkers such as lactate concentration might help to further explain the findings—on the other hand, male sex was independently associated with increased mortality after correction for baseline variables including SOFA score which integrates clinical findings and laboratory values^25^. Also, other important biomarkers such as serum levels of albumin and blood urine nitrogen could contribute to the observed sex specific differences in outcome, but were not available for this dataset, which is a limitation^26^. Third, this cohort of VIPs was not designed to evaluate gender-related differences and, therefore, this analysis remains of retrospective and thesis-generating character per se. Fourth, other potential confounders, such as smoking status or socioeconomic data are lacking, which is another limitation^27^. Fifth, we observed a sex-specific difference in 30-day-mortality, but not in ICU mortality. We speculate, that this could be due to sex-specific differences in management and treatment after discharge from ICU. However, we do not have any data available to support this notion, which, therefore, remains speculative. As the overall event rate increases from ICU mortality to the 30-day-mortality increases, sex-specific outcome differences could be present at ICU discharge, but our dataset be underpowered to detect these differences. Still, to our knowledge, this study constitutes the largest cohort of VIPs reporting gender-related outcomes. Therefore, we think that this strong signal towards adverse outcomes in male VIPs must be taken seriously.

Several biological and non-biological factors could influence gender-related outcomes. Males and females are known to differ in genetic, endocrine, and immunological factors^13,28^. Sex-specific treatment algorithms and ICU management could contribute to minimizing the observed gender disparities in VIPs. Further, male and female patients could differ in post-ICU discharge factors. Socioeconomic factors beyond the scope of this study could influence outcomes^29^. Males and females are known to differ in their readiness to assume risk and especially after an ICU stay, gender-specific complications such as falls might in part explain observed distinct outcomes^30,31^. This notion is supported by the finding that although mortality was consistently higher in males, ICU-mortality was similar between males and females, both in the unadjusted and adjusted cohorts. The benefit of intensive care in VIP is controversial in general ^32^. VIPs are known to suffer from high mortality after surviving the initial ICUstay^32^. Based on our data, male patients might be particularly prone to die after ICU discharge as ICU mortality was similar between genders, but 30-day-mortality independently associated with male gender. This finding could have several implications. First, male gender could be interpreted as an independent risk factor and influence management decisions. Second, if male VIPs are admitted to ICU and survive, post-ICU management could be particularly important in male patients. Specific geriatric ICUs and discharge to specialist geriatric wards, as well as close interdisciplinary collaboration with social workers and integration of the patient’s family, could further improve outcomes in both genders, but especially males. Therefore, not only gender-specific ICU treatment but also post-ICU management could help to improve outcomes in general and reduce observed gender disparities in VIPs.

## Conclusion

Outcomes of elderly intensive care patients evidenced independent sex differences. Male sex was associated with adverse 30-day-mortality but not ICU-mortality. Further research to identify potential sex-specific risk factors after ICU discharge is warranted.

## Supplementary information

Supplementary Figure 1.

Supplementary Information.

## Data Availability

No additional data available. All data relevant for this study will be given by the authors upon specific request.
