# Publisher Correction: Sex-specific outcome disparities in very old patients admitted to intensive care medicine: a propensity matched analysis

**DOI:** 10.1038/s41598-021-93448-6

**Published:** 2021-07-05

**Authors:** Bernhard Wernly, Raphael Romano Bruno, Malte Kelm, Ariane Boumendil, Alessandro Morandi, Finn H. Andersen, Antonio Artigas, Stefano Finazzi, Maurizio Cecconi, Steffen Christensen, Loredana Faraldi, Michael Lichtenauer, Johanna M. Muessig, Brian Marsh, Rui Moreno, Sandra Oeyen, Christina Agvald Öhman, Bernado Bollen Pinto, Ivo W. Soliman, Wojciech Szczeklik, David Niederseer, Andreas Valentin, Ximena Watson, Susannah Leaver, Carole Boulanger, Sten Walther, Joerg C. Schefold, Michael Joannidis, Yuriy Nalapko, Muhammed Elhadi, Jesper Fjølner, Tilemachos Zafeiridis, Dylan W. De Lange, Bertrand Guidet, Hans Flaatten, Christian Jung

**Affiliations:** 1 Department of Cardiology, Paracelsus Medical University, Salzburg, Austria; 2 Division of Cardiology, Department of Medicine, Karolinska Institutet, Karolinska University Hospital, Stockholm, Sweden; 3 Department of Cardiology, Pulmonology and Angiology, University Hospital, Moorenstraße 5, 40225 Duesseldorf, Germany; 4 Service de Réanimation Médicale, Publique-Hôpital de Paris, Hôpital Saint-Antoine, 75012 Paris, France; 5 Department of Rehabilitation, Hospital Ancelle Di Cremona, Cremona, Italy; 6 Geriatric Research Group, Brescia, Italy; 7 Department of Anaesthesia and Intensive Care, Ålesund Hospital, Ålesund, Norway; 8 Department of Circulation and Medical Imaging, NTNU, Trondheim, Norway; 9 Department of Intensive Care Medecine, CIBER Enfermedades Respiratorias, Corporacion Sanitaria Universitaria Parc Tauli, Autonomous University of Barcelona, Sabadell, Spain; 10 Department of Intensive Care Medecine, University Hospitals Sagrado Corazón and General de Catalunya, Quirón Salud, Barcelona-Sant Cugat, Spain; 11 Dipartimento Di Epidemiologia Clinica, IRCCS Istituto Di Ricerche Farmacologiche “Mario Negri”, Ranica, BG Italy; 12 Department of Anaesthesia IRCCS, Instituto Clínico Humanitas, Humanitas University, Milan, Italy; 13 Department of Anaesthesia and Intensive Care Medicine, Aarhus University Hospital, Aarhus, Denmark; 14 Grande Ospedale Metropolitano Niguarda, Milan, Italy; 15 Misericordiae University Hospital, Dublin, Ireland; 16 Centro Hospitalar Universitário de Lisboa Central, Nova Médical School, Faculdade de Ciências Médicas de Lisboa, Unidade de Cuidados Intensivos Neurocríticos E Trauma, Hospital de São José, Lisbon, Portugal; 17 Department of Intensive Care 1K12IC, Ghent University Hospital, Ghent, Belgium; 18 Karolinska University Hospital, Stockholm, Sweden; 19 Geneva University Hospitals, Geneva, Switzerland; 20 Department of Intensive Care Medicine, University Medical Center, University Utrecht, Utrecht, The Netherlands; 21 Intensive Care and Perioperative Medicine Division, Jagiellonian University Medical College, Kraków, Poland; 22 Department of Cardiology, University Heart Center Zurich, University Hospital Zurich, University of Zurich, Zurich, Switzerland; 23 Kardinal Schwarzenberg Hospital, Schwarzach, Austria; 24 St George’s University Hospital, London, UK; 25 Research Lead Critical Care Directorate St George’s Hospital, London, UK; 26 NAHP Section ESICM,Intensive Care Unit, Royal Devon & Exeter NHS Foundation Trust, Exeter, UK; 27 Linkoping University Hospital, Linkoping, Sweden; 28 Inselspital, Bern University Hospital, Bern, Switzerland; 29 Division of Intensive Care and Emergency Medicine, Department of Internal Medicine, Medical University Innsbruck, Innsbruck, Austria; 30 European Wellness International, ICU, Luhansk, Ukraine; 31 Alkhums Hospital, ICU, Tripoli, Libya; 32 Department of Intensive Care, Aarhus University Hospital, Aarhus, Denmark; 33 Intensive Care Unit General Hospital of Larissa Tsakalof Larissa, Larissa, Greece; 34 Service de Réanimation, INSERM, Institut Pierre Louis d’Epidémiologie et de Santé Publique, AP-HP, Sorbonne Universités, 75013 Paris, France; 35 UMR_S 1136, Institut Pierre Louis D’Epidémiologie Et de Santé Publique, INSERM, 75013 Paris, France; 36 Department of Clinical Medecine, University of Bergen, Bergen, Norway; 37 Department of Anaestesia and Intensive Care, Haukeland University Hospital, Bergen, Norway

Correction to: *Scientific Reports* 10.1038/s41598-020-74910-3, published online 29 October 2020

In the original version of this Article, the VIP2 Study Group was incorrectly listed as an author in the author list. All members of the VIP2 Study Group are listed in the accompanying Supplementary Information file.

The error has been corrected in the PDF and HTML versions of the Article, and in the Supplementary Information files.

