# Supplementary material for: Sex-specific outcome disparities in very old patients admitted to intensive care medicine: a propensity matched analysis
Source: Sci Rep. 2020 Oct 29;10:18671. doi: 10.1038/s41598-020-74910-3 (PMC7596065; doi:10.1038/s41598-020-74910-3)

Sex-specific outcome disparities in very old patients admitted to intensive care medicine: a propensity matched analysis

Bernhard Wernly MD 1; Raphael Romano Bruno MD 2; Malte Kelm MD 3; Ariane Boumendil MD 4; Alessandro Morandi MD 5; Finn H. Andersen MD 6; Antonio Artigas MD 7; Stefano Finazzi MD 8; Maurizio Cecconi MD 9; Steffen Christensen MD 10; Loredana Faraldi MD 11; Michael Lichtenauer MD 12; Johanna M Muessig MD 13; Brian Marsh MD 14; Rui Moreno MD 15; Sandra Oeyen MD 16; Christina Agvald Öhman MD 17; Bernado Bollen Pinto MD 18; Ivo W Soliman MD 19; Wojciech Szczeklik MD 20; David Niederseer PhD 21; Andreas Valentin MD 22; Ximena Watson MD 23; Susannah Leaver MD 24, Carole Boulanger MD 25, Sten Walther MD 26, Joerg C. Schefold MD 27, Michael Joannidis MD 28, Yuriy Nalapko MD 29, Muhammed Elhadi MD 30, Jesper Fjølner MD 31, Tilemachos Zafeiridis MD 32; Dylan W. De Lange MD 33, Bertrand Guidet MD 34; Hans Flaatten MD 35; Christian Jung PhD 36;

**Affiliations**

**Bernhard Wernly**, MD, ([bernhard@wernly.at](mailto:bernhard@wernly.at))

- 1. Department of Cardiology, Paracelsus Medical University, Salzburg, Austria
  2. Division of Cardiology, Department of Medicine, Karolinska Institutet, Karolinska University Hospital, Stockholm, Sweden

1. **Raphael Romano Bruno** MD, Dep. of Cardiology, Pulmonology and Angiology, University Hospital, Düsseldorf, Germany (raphael.bruno@med.uni-duesseldorf.de)
2. **Malte Kelm**, MD, Dep. of Cardiology, Pulmonology and Angiology, University Hospital, Düsseldorf, Germany (malte.kelm@med.uni-duesseldorf.de)
3. **Ariane Boumendil**, PhD. Assistance Publique-Hôpital de Paris, Hôpital Saint-Antoine, Service de Réanimation Médicale, Paris, F-75012, France ([ariane.boumendil@gmail.com](mailto:ariane.boumendil@gmail.com))
4. **Alessandro Morandi**, MD MPH. Department of Rehabilitation Hospital Ancelle di Cremona, Italy; Geriatric Research Group, Brescia, Italy ([morandi.alessandro@gmail.com](mailto:morandi.alessandro@gmail.com))
5. **Finn H. Andersen**, MD PhD, Dep. Of Anaesthesia and Intensive Care, Ålesund Hospital, Ålesund, Norway. NTNU, Dep of Circulation and Medical Imaging, Trondheim, Norway (finn.andersen@ntnu.no)
6. **Antonio Artigas**, MD PhD. ([aartigas@tauli.cat](mailto:aartigas@tauli.cat))
   1. Department of Intensive Care Medecine, CIBER Enfermedades Respiratorias, Corporacion Sanitaria Universitaria Parc Tauli, Autonomous University of Barcelona, Sabadell, Spain
   2. Department of Intensive Care Medecine, University Hospitals Sagrado Corazón and General de Catalunya. Quirón Salud. Barcelona-Sant Cugat, Spain.
7. **Stefano Finazzi**, MD Dipartimento di Epidemiologia Clinica, IRCCS Istituto di Ricerche Farmacologiche "Mario Negri", Ranica, BG, Italy (stefano.finazzi@marionegri.it)
8. **Maurizio Cecconi**, MD Department of Anaesthesia IRCCS Instituto Clínico Humanitas, Humanitas University, Milan, Italy (Maurizio.cecconi@huamitas.it)
9. **Steffen Christensen**, MD PhD. Department of Anaesthesia and Intensive Care Medicine, Aarhus University Hospital, Denmark (steffen.christensen@auh.rm.dk)
10. **Loredana Faraldi** MD ASST Grande Ospedale Metropolitano Niguarda, Milano, Italy (loredana.faraldi@ospedaleniguarda.it)
11. **Michael Lichtenauer**, PhD, Department of Cardiology, Paracelsus Medical University, Salzburg, Austria ([m.lichtenauer@salk.at](mailto:m.lichtenauer@salk.at))
12. **Johanna M Muessig** MD, Dep. of Cardiology, Pulmonology and Angiology, University Hospital, Düsseldorf, Germany ([johanna.muessig@med.uni-duesseldorf.de](mailto:johanna.muessig@med.uni-duesseldorf.de))
13. **Brian Marsh**, MD Mater Misericordiae University Hospital, Dublin, Ireland (bmarsh@mater.ie)
14. **Rui Moreno,** MD, PhD, Unidade de Cuidados Intensivos Neurocríticos e Trauma. Hospital de São José, Centro Hospitalar Universitário de Lisboa Central, Faculdade de Ciências Médicas de Lisboa, Nova Médical School, Lisbon, Portugal (r.moreno@mail.telepac.pt)
15. **Sandra Oeyen**, MD. Department of Intensive Care 1K12IC Ghent University Hospital, Ghent, Belgium (Sandra.Oeyen@UGent.be)
16. **Christina Agvald Öhman** MD PhD. Karolinska University Hospital, Sweden (christina.agvald-ohman@sll.se)
17. **Bernardo Bollen Pinto** MD Geneva University Hospitals, Geneva, Switzerland (bollenpinto@gmail.com)
18. **Ivo W Soliman**, MD. Department of Intensive Care Medicine, University Medical Center, University Utrecht, Utrecht, the Netherlands, (i.w.soliman@umcutrecht.nl)
19. **Wojciech Szczeklik** MD, PhD, Intensive Care and Perioperative Medicine Division, Jagiellonian University Medical College, Kraków, Poland ([wojciech.szczeklik@uj.edu.pl](mailto:wojciech.szczeklik@uj.edu.pl))
20. **David Niederseer** MD, PhD, Department of Cardiology, University Heart Center Zurich, University Hospital Zurich, University of Zurich, Zurich, Switzerland (david.niederseer@usz.ch)
21. **Andreas Valentin** MD Kardinal Schwarzenberg Hospital, Schwarzach, Austria (Andreas.Valentin@ks-klinikum.at)
22. **Ximena Watson** MD. St George’s University Hospital, London, UK (ugm2xw@doctors.org.uk)
23. **Susannah Leaver** MD. Research Lead Critical Care Directorate St George’s Hospital, London, UK (susannahleaver@nhs.net)
24. **Carole Boulanger** MD. Chair NAHP Section ESICM,Intensive Care Unit, Royal Devon & Exeter NHS Foundation Trust, Exeter, UK (carole.boulanger@nhs.net)
25. **Sten Walther** MD. Linkoping University Hospital, Linkoping, Sweden (sten.walther@ki.se)
26. **Joerg C. Schefold** MD. Inselspital, Bern University Hospital Bern, CH (joerg.schefold@insel.ch)
27. **Michael Joannidis** MD. Division of Intensive Care and Emergency Medicine, Department of Internal Medicine, Medical University Innsbruck, Innsbruck, Austria (Michael.joannidis@i-med.ac.at)
28. **Yuriy Nalapko** MD. European Wellness International, ICU, Luhansk, Ukraine (nalapko@ukr.net)
29. **Muhammed Elhadi** MD Alkhums Hospital, ICU, Tripoli, Libya.
30. **Jesper Fjølner** MD Department of Intensive Care, Aarhus University Hospital, Aarhus, Denmark (jespfjoe@rm.dk)
31. **Tilemachos Zafeiridis** MD, Intensive Care Unit General Hospital of Larissa Tsakalof Larissa, Greece ([tilemachos@hotmail.com](mailto:tilemachos@hotmail.com))
32. **Dylan W. De Lange,** MD PhD. Department of Intensive Care Medicine, University Medical Center, University Utrecht, the Netherlands (d.w.delange@umcutrecht.nl)
33. **Bertrand Guidet**, MD.
    1. Assistance Publique - Hôpitaux de Paris, Hôpital Saint-Antoine, service de réanimation médicale, Paris, F-75012, France ([bertrand.guidet@aphp.fr](mailto:bertrand.guidet@aphp.fr)).
    2. Sorbonne Universités, UPMC Univ Paris 06, UMR_S 1136, Institut Pierre Louis d’Epidémiologie et de Santé Publique, F-75013, Paris, France.
    3. INSERM, UMR_S 1136, Institut Pierre Louis d’Epidémiologie et de Santé Publique, F-75013, Paris, France
34. **Hans Flaatten**, MD PhD. Department of Clinical Medecine,University of Bergen, Department of Anaestesia and Intensive Care, Haukeland University Hospital , Bergen, Norway (hans.flaatten@uib.no)
35. **Christian Jung**, MD PhD, Dep. of Cardiology, Pulmonology and Angiology, University Hospital, Düsseldorf, Germany ([Christian.Jung@med.uni-duesseldorf.de](mailto:Christian.Jung@med.uni-duesseldorf.de))

Corresponding author: Prof. Christian Jung, M.D. PhD

Division of Cardiology, Pulmonology, and Vascular Medicine

University Duesseldorf

Moorenstraße 5

40225 Duesseldorf

Germany

Phone: Telefon: +49 211 81-00

Email: christian.jung@med.uni-duesseldorf.de


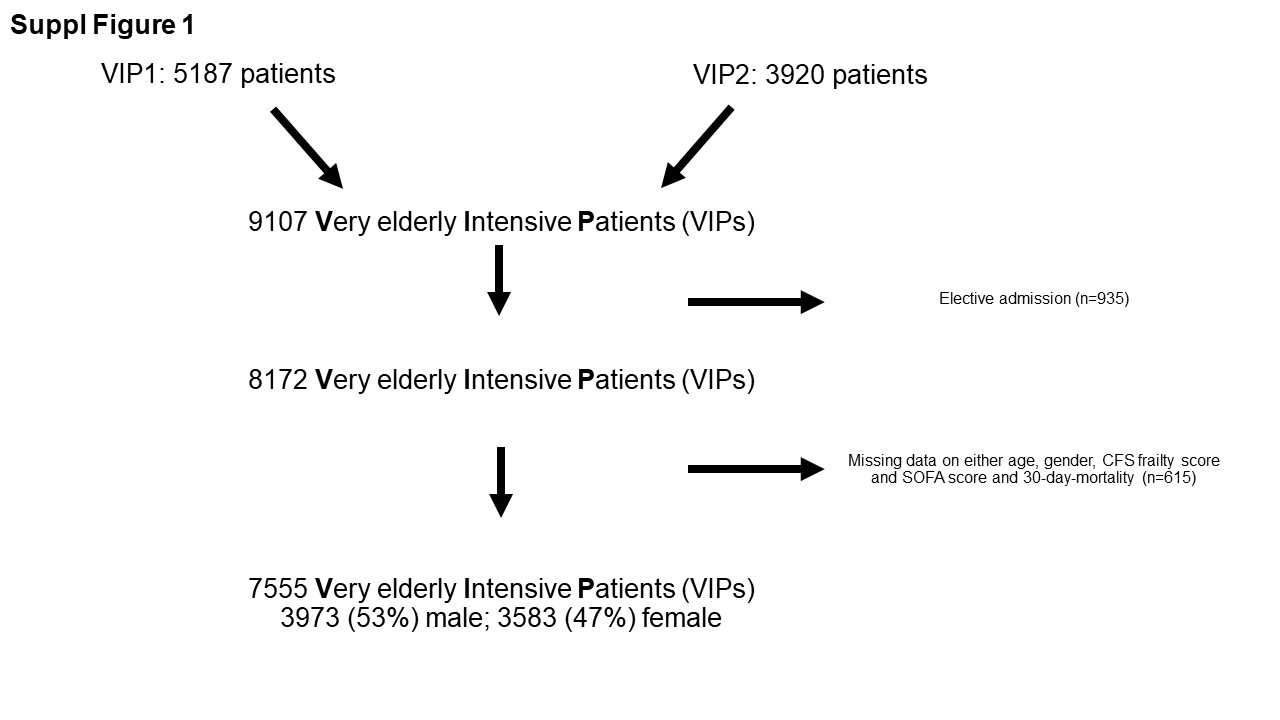

Supplement: Supplementary file 1 — Supplementary Figure 1. [file 41598_2020_74910_MOESM1_ESM.docx]
