# Supplementary material for: Sex-specific outcome disparities in very old patients admitted to intensive care medicine: a propensity matched analysis
Source: Sci Rep. 2020 Oct 29;10:18671. doi: 10.1038/s41598-020-74910-3 (PMC7596065; doi:10.1038/s41598-020-74910-3)
Supplement: Supplementary file 2 — Supplementary Information. [file 41598_2020_74910_MOESM2_ESM.docx]

Sex-specific outcome disparities in very old patients admitted to intensive care medicine: a propensity matched analysis

Bernhard Wernly MD 1; Raphael Romano Bruno MD 2; Malte Kelm MD 3; Ariane Boumendil MD 4; Alessandro Morandi MD 5; Finn H. Andersen MD 6; Antonio Artigas MD 7; Stefano Finazzi MD 8; Maurizio Cecconi MD 9; Steffen Christensen MD 10; Loredana Faraldi MD 11; Michael Lichtenauer MD 12; Johanna M Muessig MD 13; Brian Marsh MD 14; Rui Moreno MD 15; Sandra Oeyen MD 16; Christina Agvald Öhman MD 17; Bernado Bollen Pinto MD 18; Ivo W Soliman MD 19; Wojciech Szczeklik MD 20; David Niederseer PhD 21; Andreas Valentin MD 22; Ximena Watson MD 23; Susannah Leaver MD 24, Carole Boulanger MD 25, Sten Walther MD 26, Joerg C. Schefold MD 27, Michael Joannidis MD 28, Yuriy Nalapko MD 29, Muhammed Elhadi MD 30, Jesper Fjølner MD 31, Tilemachos Zafeiridis MD 32; Dylan W. De Lange MD 33, Bertrand Guidet MD 34; Hans Flaatten MD 35; Christian Jung PhD 36;

**Affiliations**

**Bernhard Wernly**, MD, ([bernhard@wernly.at](mailto:bernhard@wernly.at))

- 1. Department of Cardiology, Paracelsus Medical University, Salzburg, Austria
  2. Division of Cardiology, Department of Medicine, Karolinska Institutet, Karolinska University Hospital, Stockholm, Sweden

1. **Raphael Romano Bruno** MD, Dep. of Cardiology, Pulmonology and Angiology, University Hospital, Düsseldorf, Germany (raphael.bruno@med.uni-duesseldorf.de)
2. **Malte Kelm**, MD, Dep. of Cardiology, Pulmonology and Angiology, University Hospital, Düsseldorf, Germany (malte.kelm@med.uni-duesseldorf.de)
3. **Ariane Boumendil**, PhD. Assistance Publique-Hôpital de Paris, Hôpital Saint-Antoine, Service de Réanimation Médicale, Paris, F-75012, France ([ariane.boumendil@gmail.com](mailto:ariane.boumendil@gmail.com))
4. **Alessandro Morandi**, MD MPH. Department of Rehabilitation Hospital Ancelle di Cremona, Italy; Geriatric Research Group, Brescia, Italy ([morandi.alessandro@gmail.com](mailto:morandi.alessandro@gmail.com))
5. **Finn H. Andersen**, MD PhD, Dep. Of Anaesthesia and Intensive Care, Ålesund Hospital, Ålesund, Norway. NTNU, Dep of Circulation and Medical Imaging, Trondheim, Norway (finn.andersen@ntnu.no)
6. **Antonio Artigas**, MD PhD. ([aartigas@tauli.cat](mailto:aartigas@tauli.cat))
   1. Department of Intensive Care Medecine, CIBER Enfermedades Respiratorias, Corporacion Sanitaria Universitaria Parc Tauli, Autonomous University of Barcelona, Sabadell, Spain
   2. Department of Intensive Care Medecine, University Hospitals Sagrado Corazón and General de Catalunya. Quirón Salud. Barcelona-Sant Cugat, Spain.
7. **Stefano Finazzi**, MD Dipartimento di Epidemiologia Clinica, IRCCS Istituto di Ricerche Farmacologiche "Mario Negri", Ranica, BG, Italy (stefano.finazzi@marionegri.it)
8. **Maurizio Cecconi**, MD Department of Anaesthesia IRCCS Instituto Clínico Humanitas, Humanitas University, Milan, Italy (Maurizio.cecconi@huamitas.it)
9. **Steffen Christensen**, MD PhD. Department of Anaesthesia and Intensive Care Medicine, Aarhus University Hospital, Denmark (steffen.christensen@auh.rm.dk)
10. **Loredana Faraldi** MD ASST Grande Ospedale Metropolitano Niguarda, Milano, Italy (loredana.faraldi@ospedaleniguarda.it)
11. **Michael Lichtenauer**, PhD, Department of Cardiology, Paracelsus Medical University, Salzburg, Austria ([m.lichtenauer@salk.at](mailto:m.lichtenauer@salk.at))
12. **Johanna M Muessig** MD, Dep. of Cardiology, Pulmonology and Angiology, University Hospital, Düsseldorf, Germany ([johanna.muessig@med.uni-duesseldorf.de](mailto:johanna.muessig@med.uni-duesseldorf.de))
13. **Brian Marsh**, MD Mater Misericordiae University Hospital, Dublin, Ireland (bmarsh@mater.ie)
14. **Rui Moreno,** MD, PhD, Unidade de Cuidados Intensivos Neurocríticos e Trauma. Hospital de São José, Centro Hospitalar Universitário de Lisboa Central, Faculdade de Ciências Médicas de Lisboa, Nova Médical School, Lisbon, Portugal (r.moreno@mail.telepac.pt)
15. **Sandra Oeyen**, MD. Department of Intensive Care 1K12IC Ghent University Hospital, Ghent, Belgium (Sandra.Oeyen@UGent.be)
16. **Christina Agvald Öhman** MD PhD. Karolinska University Hospital, Sweden (christina.agvald-ohman@sll.se)
17. **Bernardo Bollen Pinto** MD Geneva University Hospitals, Geneva, Switzerland (bollenpinto@gmail.com)
18. **Ivo W Soliman**, MD. Department of Intensive Care Medicine, University Medical Center, University Utrecht, Utrecht, the Netherlands, (i.w.soliman@umcutrecht.nl)
19. **Wojciech Szczeklik** MD, PhD, Intensive Care and Perioperative Medicine Division, Jagiellonian University Medical College, Kraków, Poland ([wojciech.szczeklik@uj.edu.pl](mailto:wojciech.szczeklik@uj.edu.pl))
20. **David Niederseer** MD, PhD, Department of Cardiology, University Heart Center Zurich, University Hospital Zurich, University of Zurich, Zurich, Switzerland (david.niederseer@usz.ch)
21. **Andreas Valentin** MD Kardinal Schwarzenberg Hospital, Schwarzach, Austria (Andreas.Valentin@ks-klinikum.at)
22. **Ximena Watson** MD. St George’s University Hospital, London, UK (ugm2xw@doctors.org.uk)
23. **Susannah Leaver** MD. Research Lead Critical Care Directorate St George’s Hospital, London, UK (susannahleaver@nhs.net)
24. **Carole Boulanger** MD. Chair NAHP Section ESICM,Intensive Care Unit, Royal Devon & Exeter NHS Foundation Trust, Exeter, UK (carole.boulanger@nhs.net)
25. **Sten Walther** MD. Linkoping University Hospital, Linkoping, Sweden (sten.walther@ki.se)
26. **Joerg C. Schefold** MD. Inselspital, Bern University Hospital Bern, CH (joerg.schefold@insel.ch)
27. **Michael Joannidis** MD. Division of Intensive Care and Emergency Medicine, Department of Internal Medicine, Medical University Innsbruck, Innsbruck, Austria (Michael.joannidis@i-med.ac.at)
28. **Yuriy Nalapko** MD. European Wellness International, ICU, Luhansk, Ukraine (nalapko@ukr.net)
29. **Muhammed Elhadi** MD Alkhums Hospital, ICU, Tripoli, Libya.
30. **Jesper Fjølner** MD Department of Intensive Care, Aarhus University Hospital, Aarhus, Denmark (jespfjoe@rm.dk)
31. **Tilemachos Zafeiridis** MD, Intensive Care Unit General Hospital of Larissa Tsakalof Larissa, Greece ([tilemachos@hotmail.com](mailto:tilemachos@hotmail.com))
32. **Dylan W. De Lange,** MD PhD. Department of Intensive Care Medicine, University Medical Center, University Utrecht, the Netherlands (d.w.delange@umcutrecht.nl)
33. **Bertrand Guidet**, MD.
    1. Assistance Publique - Hôpitaux de Paris, Hôpital Saint-Antoine, service de réanimation médicale, Paris, F-75012, France ([bertrand.guidet@aphp.fr](mailto:bertrand.guidet@aphp.fr)).
    2. Sorbonne Universités, UPMC Univ Paris 06, UMR_S 1136, Institut Pierre Louis d’Epidémiologie et de Santé Publique, F-75013, Paris, France.
    3. INSERM, UMR_S 1136, Institut Pierre Louis d’Epidémiologie et de Santé Publique, F-75013, Paris, France
34. **Hans Flaatten**, MD PhD. Department of Clinical Medecine,University of Bergen, Department of Anaestesia and Intensive Care, Haukeland University Hospital , Bergen, Norway (hans.flaatten@uib.no)
35. **Christian Jung**, MD PhD, Dep. of Cardiology, Pulmonology and Angiology, University Hospital, Düsseldorf, Germany ([Christian.Jung@med.uni-duesseldorf.de](mailto:Christian.Jung@med.uni-duesseldorf.de))

Corresponding author: Prof. Christian Jung, M.D. PhD

Division of Cardiology, Pulmonology, and Vascular Medicine

University Duesseldorf

Moorenstraße 5

40225 Duesseldorf

Germany

Phone: Telefon: +49 211 81-00

Email: christian.jung@med.uni-duesseldorf.de

VIP2 study contributors:

| **Hospital** | **City** | **ICU** | **Name** |
| --- | --- | --- | --- |

**Austria**

| Medical University Innsbruck | Innsbruck | Division of Intensive Care and Emergency Medicine, Department of Internal Medicine | Michael Joannidis |
| --- | --- | --- | --- |
| Medical University Graz | Graz | Allgemeine Medizin Intensivstation | Philipp Eller |
| Medical University of Innsbruck | Innsbruck | Department of Neurology, Neurocritical Care Unit | Raimund Helbok |
| Hospital of St. John of God | Vienna | ICU B5 | René Schmutz |

**Belgium**

| AZ Maria Middelares Ghent | Ghent | Department of Intensive Care | Joke Nollet |
| --- | --- | --- | --- |
| OLVrouw Hospital Aalst | Aalst | Department of Intensive Care | Nikolaas de Neve |
| AZ Sint-Lucas | Ghent | Department of Intensive Care | Pieter De Buysscher |
| Ghent University Hospital | Ghent | Department of Intensive Care | Sandra Oeyen |
| AZ Sint-Blasius | Dendermonde | Department of Intensive Care | Walter Swinnen |

**Croatia**

| Clinical Hospital Centre Split | Split | Institute for Intensive Medicine | Marijana Mikačić |
| --- | --- | --- | --- |

**Denmark**

| Bispebjerg Hospital | Copenhagen | Intensiv Terapi Afsnit | Anders Bastiansen |
| --- | --- | --- | --- |
| Regionshospitalet Randers | Randers | ITA | Andreas Husted |
| Sygehus Lillebælt, Kolding Sygehus | Kolding | Bedøvelse og Intensiv | Bård E. S. Dahle |
| Aarhus University Hospital | Aarhus | Intensive Care East | Christine Cramer |
| Viborg Regional Hospital | Viborg | Department of Anaesthesiology and Intensive Care | Christoffer Sølling |
| Nordsjællands Hospital, University of Copenhagen | Hillerød | Department of Anaesthesiology and Intensive Care | Dorthe Ørsnes |
| Regions Hospital Herning | Herning | Intensiv Herning | Jakob Edelberg Thomsen |
| Vejle | Vejle | A710 Vejle | Jonas Juul Pedersen |
| Regionshospital Nordjylland Hjørring | Hjørring | Intensiv | Mathilde Hummelmose Enevoldsen |
| Aarhus University Hospital | Aarhus | Intensive Care North | Thomas Elkmann |

**England**

| Yeovil District Hospital | Yeovil | Intensive Care Unit | Agnieszka Kubisz-Pudelko |
| --- | --- | --- | --- |
| Peterborough City Hospital | Petersborough | Critical Care Unit | Alan Pope |
| Queen Elizabeth Hospital | London | Critical Care Queen Elizabeth Hospital | Amy Collins |
| Croydon University Hospital | Croydon | Croydon University Hospital ITU | Ashok S. Raj |
| Royal Devon & Exeter NHS Foundation Trust | Exeter | Intensive Care Unit | Carole Boulanger |
| South Tyneside District Hospital | South Shields | ITU | Christian Frey |
| Maidstone | Maidstone | Intensive Care/High Dependency | Ciaran Hart |
| University Hospital Southampton | Southampton | General Intensive Care Unit | Clare Bolger |
| St George´s University Hospitals NHS Foundation trust | London | Cardiothoracic Intensive Care Unit (CTICU) | Dominic Spray |
| Norfolk and Norwich University Hospital | Norwich | Critical care complex | Georgina Randell |
| Royal Free Hospital NHS Foundation Trust | London | ICU 4 | Helder Filipe |
| Royal Liverpool University Hospital | Liverpool | Intensive care | Ingeborg D Welters |
| Royal Hampshire County Hospital | Winchester | ICU | Irina Grecu |
| St George´s University Hospitals NHS Foundation trust | London | Acute Dependency Unit | Jane Evans |
| Blackpool Victoria Hospital | Blackpool | General Critical Care Unit | Jason Cupitt |
| Worthing Hospital | Worthing | ICU | Jenny Lord |
| James Cook University Hospital | Midlesbrough | ICU 2 | Jeremy Henning |
| Tunbridge Wells Hospital | Pembury | Intensive care unit | Joanne Jones |
| St George´s University Hospitals NHS Foundation trust | London | Neuro Intensive Care | Jonathan Ball |
| James Paget University Hospital | Norfolk | ICU/HDU | Julie North |
| Royal Papworth Hospital NHS Foundation Trust | Cambridge | ICU | Kiran Salaunkey |
| Royal Sussex County Hospital | Brighton | Level 7 | Laura Ortiz-Ruiz De Gordoa |
| Salisbury | Salisbury | Radnor | Louise Bell |
| Royal Bolton Hospital | Bolton | Royal Bolton CRITICAL CARE | Madhu Balasubramaniam |
| Chelsea and Westminster Hospital | London | Adult Intensive Care Unit | Marcela Vizcaychipi |
| Countess of Chester Hospital | Chester | Intensive Care Unit | Maria Faulkner |
| Hampshire Hospitals Foundation Trust | Basingstoke | Basingstoke and North Hampshire Hospital | McDonald Mupudzi |
| Hinchingbrooke Hospital | Huntingdon | Critical Care | Megan Lea-Hagerty |
| Russells Hall Hospital | Dudley | Intensive Care Unit Russells Hall | Michael Reay |
| Royal Cornwall Hospital Trust | Cornwall | Critical Care Unit | Michael Spivey |
| Northern Devon Healthcare NHS Trust | Barnstaple | North Devon District Hospital | Nicholas Love |
| Chesterfield Royal Hospital | Chesterfield | Intensive Care Unit | Nick Spittle |
| Royal Bournemouth Hospital | Bournemouth | Bournemouth Critical Care Unit | Nigel White |
| Dorset County | Dorchester | ICU DCH | Patricia Williams |
| Surrey and Sussex Healthcare NHS Trust | Redhill | East Surrey Hospital | Patrick Morgan |
| Darent Valley | Dartford | ICU | Phillipa Wakefield |
| Royal Surrey County Hospital | Guildford | Royal Surrey | Rachel Savine |
| Wirral University Teaching Hospital | Birkenhead | Critical care | Reni Jacob |
| Musgrove Park Hospital | Taunton | Critical care Unit | Richard Innes |
| Kent and Canterbury Hospital | Canterbury | K&C ITU | Ritoo Kapoor |
| West Suffolk NHS Foundation Trust | Bury St Edmunds | Critical Care | Sally Humphreys |
| QAH | Portsmouth | Dept Critical Care QAH (DCCQ) | Steve Rose |
| Whiston Hospital | Liverpool | Ward 4E | Susan Dowling |
| St George´s University Hospitals NHS Foundation trust | London | General Intensive care | Susannah Leaver |
| North Tees University Hospital | Stockton on Tees | Critical Care Unit | Tarkeshwari Mane |
| Bradford Teaching Hospitals NHS Foundation Trust | Bradford | Bradford Royal Infirmary | Tom Lawton |
| Medway Maritime Hospital | Medway | Adult Intensive Care Unit | Vongayi Ogbeide |
| University Hospital Lewisham | Lewisham | ICU/HDU Lewisham | Waqas Khaliq |
| St Richards Hospital | Chichester | Itchenor | Yolanda Baird |

**France**

| CH Francois Mitterand | Pau | Reanimation polyvalente | Antoine Romen |
| --- | --- | --- | --- |
| Hôpital Privé Claude Galien | Quincy sous Sénart | Polyvalente | Arnaud Galbois |
| Saint Antoine | Paris | Medecine Intensive Reanimation | Bertrand Guidet |
| Germon and Gauthier | Béthune | Médecine Intensive Réanimation | Christophe Vinsonneau |
| Hôpital Ambroise Paré | Boulogne Billancourt | Medecine Intensive Reanimation | Cyril Charron |
| CH Dr Schaffner | Lens | Reanimation polyvalente | Didier Thevenin |
| Hopital Européen Georges Pompidou | Paris | Médecine Intensive Réanimation | Emmanuel Guerot |
| CHU de Besançon | Besançon | Département de Anesthésie Réanimation Chirurgicale | Guillaume Besch |
| Hôpital Cochin | Paris | Médecine Intensive Réanimation | Guillaume Savary |
| Victor Dupouy | Argenteuil | Service de Réanimation Polyvalente et USC | Hervé Mentec |
| Centre Hospitalier Général | Cambrai | Réanimation polyvalente | Jean-Luc Chagnon |
| Dieppe General Hospital | Dieppe | Médecine Intensive Réanimation | Jean-Philippe Rigaud |
| CHU Dijon Bourgogne | Dijon | Medecine intensive-Réanimation | Jean-Pierre Quenot |
| CH Bigorre | Tarbes | service de réanimation polyvalente | Jeremy Castanera |
| CH de Charleville-Mézières | Charleville-Mezieres | Medecine Intensive Reanimation | Jérémy Rosman |
| CHU Amiens | Amiens | Reanimaiton medicale | Julien Maizel |
| Groupe Hospitalier Paris Saint Joseph | Paris | Réanimation polyvalente | Kelly Tiercelet |
| CHU de Besançon | Besancon | Réanimation Médicale | Lucie Vettoretti |
| CH DAX | DAX | Réanimation polyvalente | Maud Mousset Hovaere |
| Louis Mourier | Colombes | Réanimation médico-chirurgicale | Jonathan Messika |
| Tenon | Paris | Service de Réanimation Médico Chirurgicale | Michel Djibré |
| Groupe Hospitalier Sud Ile de France | Melun | Département de médecine intensive | Nathalie Rolin |
| Clinique Du Millenaire | Montpellier | Reanimation Chirurugicale II et III | Philippe Burtin |
| Marne La Vallee | Jossigny | Reanimation Polyvalente | Pierre Garcon |
| CHU Lille | Lille | Critical Care Center | Saad Nseir |
| CHU de Caen | Caen | Service de Réanimation Médicale | Xavier Valette |

**Germany**

| Klinikum rechts der Isar TU München | München | Toxikologische Intensivstation | Christian Rabe |
| --- | --- | --- | --- |
| University Hospital Ulm | Ulm | Anesthesiologic Intensive Care Department | Eberhard Barth |
| Katholisches Krankenhaus St. Johann Nepomuk | Erfurt | Klinik für Innere Medizin II/ Kardiologie und Internistische Intensivmedizin | Henning Ebelt |
| Klinikum rechts der Isar, School of Medicine, Technical University of Munich | München | Intensivstation IS2/L2a | Kristina Fuest |
| Jena University Hospital, Department of Internal Medicine I | Jena | Internistische Intensivstation | Marcus Franz |
| West German Heart and Vascular Center Essen (WHGZ) | Essen | INTK | Michael Horacek |
| Universitätsmedizin der Johannes Gutenberg-Universität Mainz | Mainz | Anästhesie-Intensivstation | Michael Schuster |
| University Hospital Frankfurt | Frankfurt am Main | Department of Anaesthesiology, Intensive Care Medicine and Pain Therapy | Patrick Meybohm |
| University Hospital Düsseldorf | Düsseldorf | MI1/2 | Raphael Romano Bruno |
| Robert-Bosch-Krankenhaus | Stuttgart | 1D | Sebastian Allgäuer |
| Heidelberg University Hospital | Heidelberg | Station 13 IOPIS | Simon Dubler |
| Klinikum rechts der Isar, School of Medicine, Technical University of Munich | München | Intensivstation IS1 / M2b | Stefan J Schaller |
| University Hospital Leipzig | Leipzig | Department of Anesthesiology and Intensive Care Medicine | Stefan Schering |
| St Vincenz Hospital | Limburg/Lahn | Intensive care unit | Stephan Steiner |
| Hannover Medical School | Hannover | 44 | Thorben Dieck |
| Universitätsklinikum Knappschaftskrankenhaus Bochum | Bochum | Operative IBA | Tim Rahmel |
| Universitätsklinikum Schleswig-Holstein | Lübeck | IKI 12a | Tobias Graf |

**Greece**

| Asklepieio Voulas | Athens | ICU | Anastasia Koutsikou |
| --- | --- | --- | --- |
| Xanthi General Hospital | Xanthi | Xanthi ICU | Aristeidis Vakalos |
| Sismanoglio - Amallia Fleming G. H | Marousi - Athens Attika | Sismanoglio | Bogdan Raitsiou |
| General Hospital Agios Pavlos | Thessaloniki | ICU Agios Pavlos | Elli Niki Flioni |
| General Hospital of Larissa | Larissa | General ICU | Evangelia Neou |
| Lamia General Hospita | Lamia | Lamia ICU | Fotios Tsimpoukas |
| University Hospital of Ioannina | Ioannina | Intensive Care Unit | Georgios Papathanakos |
| General Hospital of Athens Korgialeneio Mbenakeio Red Cross | Athens | ICU | Giorgos Marinakis |
| General Hospital of Eleusis Thriassio | Eleusis | ICU Latsio | Ioannis Koutsodimitropoulos |
| KONSTANTOPOULEION GEN. HOSPITAL | Athens | General ICU | Kounougeri Aikaterini |
| Sotiria Hospital | Athens | ICU 1st Department of Pulmonary Medicine Athens Medical School, National and Kapodistrian University of Athens | Nikoletta Rovina |
| General Hospital of Patra | Achaia | ICU | Stylliani Kourelea |
| G Gennimatas Hospital of Thessaloniki | Thessaloniki | ICU G GENNIMATAS | Polychronis Tasioudis |
| Agioi Anargiroi Hospital | Athens | General ICU | Vasiiios Zidianakis |
| Theagenio | Theassaloniki | Meth Theagenio | Vryza Konstantinia |
| University General Hospital Ahepa | Thessaloniki | Metha | Zoi Aidoni |

**Ireland**

| Mater Misericordiae University Hospital | Dublin | Department of Critical Care Medicine | Brian Marsh |
| --- | --- | --- | --- |
| University Hospital Limerick | Limerick | UHL ICU | Catherine Motherway |
| University Hospital Galway | Galway | General ICU | Chris Read |
| St James´s Hospital | Dublin | ICU | Ignacio Martin-Loeches |

**Italy**

| Arnas Ospedale Civico De Christina Benfratelli | Palermo | Terapia Intensiva Polivalente Con Trauma Center | Andrea Neville Cracchiolo |
| --- | --- | --- | --- |
| Istituto Ortopedico Rizzoli | Bologna | TIPO | Aristide Morigi |
| San Giuseppe | Empoli | Terapia Intensiva | Italo Calamai |
| Humanitas Reseach Hospital | Milan | General ICU | Stefania Brusa |

**Libya**

| Al-Zawia University Hospital | Al-Zawia | ICU | Ahmed Elhadi |
| --- | --- | --- | --- |
| Alkhums Hospital | Alkhums | ICU | Ahmed Tarek |
| Elkhadra Hospital | Tripoli | ICU | Ala Khaled |
| Abo Selim Trauma Hospital | Tripoli | ICU | Hazem Ahmed |
| Tripoli Medical Center | Tripoli | CCU | Wesal Ali Belkhair |

**Netherland**

| Medisch Spectrum Twente | Enschede | Intensive Care Center | Alexander D. Cornet |
| --- | --- | --- | --- |
| Erasmus Medical Center | Rotterdam | ICU adults | Diederik Gommers |
| UMC Utrecht | Utrecht | ICU departement | Dylan de Lange |
| Albert Schweitzer Ziekenhuis | Dordrecht | ICU asz | Eva van Boven |
| Isala Hospital | Zwolle | Intensive Care | Jasper Haringman |
| Diakonessenhuis Utrecht | Utrecht | Intensive care | Lenneke Haas |
| Haga Ziekenhuis | The Hague | ICU | Lettie van den Berg |
| Canisius Wilhelmina Ziekenhuis | Nijmegen | C38 | Oscar Hoiting |
| Jeroen Bosch Ziekenhuis | Den Bosch | IC JBZ | Peter de Jager |
| Medical Centre Leeuwarden | Leeuwarden | Department of Intensive Care | Rik T. Gerritsen |
| Zuyderland Medical Center | Heerlen | Zuyderland Heerlen | Tom Dormans |
| University Medical Center Groningen | Groningen | Department of Critical Care | Willem Dieperink |

**Norway**

| Førde Central Hospital | Førde | Department of Emergency Medicine and Inensive Care | Alena Breidablik |
| --- | --- | --- | --- |
| Kongsberg | Kongsberg | Intensivavdelingen | Anita Slapgard |
| Sykehuset Østfold | Sarpsborg | Intensiv | Anne-Karin Rime |
| Sykehuset Telemark | Skien | Intensiv Skien | Bente Jannestad |
| Haukeland University Hospital | Bergen | General ICU | Britt Sjøbøe |
| Ålesund | Ålesund | Medisinsk intensiv | Eva Rice |
| Ålesund hospital | Ålesund | Dept. Anesthesia and Intensive Care, Surgical ICU | Finn H. Andersen |
| Kristiansund sykehus Helse Møre og Romsdal HF | Kristiansund N | Intensiv Kristiansund | Hans Frank Strietzel |
| Namsos Sykehus | Namsos | Intensivavdeling | Jan Peter Jensen |
| Haukeland University Hospital | Bergen | Medisinsk intensiv og overvåkning (MIO) | Jørund Langørgen |
| Oslo University Hospital | Oslo | Intensive Care section Ullevaal | Kirsti Tøien |
| Stavanger University Hospital | Stavanger | Department of Intensive Care | Kristian Strand |
| Haugesund sjukehus | Haugesund | Intensivavdelingen | Michael Hahn |
| St Olavs University Hospital | Oslo | Hovedintensiv | Pål Klepstad |

**Poland**

| Szpital Wojewódzki w Bełchatowie | Bełchatów | Oddział Intensywnej Terapii | Aleksandra Biernacka |
| --- | --- | --- | --- |
| Heliodor Swiecicki Clinical Hospital at the Karol Marcinkowski Medical University in Poznan | Poznań | Anaesthesiology intensive care and pain treatment Department | Anna Kluzik |
| University Hospital in Zielona Góra | Zielona Góra | Clinical Department od Anesthesiology and Intensiv Care | Bartosz Kudlinski |
| Regional Teaching Hospital | Bielsko-Biała | Department of Anaesthesiology and Intensive Care | Dariusz Maciejewski |
| St. John Grande Hospital | Kraków | Oddział Anestezjologii i Intensywnej Terapii | Dorota Studzińska |
| The John Paul II Hospital | Krakow | Department of Anesthesiology and Intensive Care | Hubert Hymczak |
| Uniwersyteckie Centrum Kliniczne w Gdańsku | Gdańsk | Klinika Anestezjologii i Intensywnej Terapii | Jan Stefaniak |
| Pomeranian Medical University | Szczecin | Department of Anesthesiology and Intensive Care | Joanna Solek-Pastuszka |
| University Hospital in Cracow | Kraków | Anaesthesiology and Intensive Care Unit No.1 | Joanna Zorska |
| Regionalne Centrum Zdrowia w Lubinie | Lubin | Oddział Anestezjologii i Intensywnej Terapii | Katarzyna Cwyl |
| University Clinical Center Katowice | Katowice | Department of Anaesthesiology and Intensive Care - School of Medicine in Katowice, Medical University of Silesia | Lukasz J. Krzych |
| Teching Hospital No 2 | Szczecin | Department Anaesthesiology Intensive Therapy and Acute Poisoning | Maciej Zukowski |
| 4th Military Hospital in Wrocław | Wrocław | Anesthesia and Intensive Care Unit | Małgorzata Lipińska-Gediga |
| Centrum Chorób Płuc | Łódź | Oddział Anestezjologii i Intensywnej Terapii | Marek Pietruszko |
| The Dr Wł. Biegański Regional Specialist Hospital in Łódź | Łódź | Department of Anaesthesiology and Intensive Therapy - Centre for Artificial Extracorporeal Kidney and Liver Support | Mariusz Piechota |
| Central Clinical Hospital CKD - University Medical College in Lodz | Łódź | Anaesthesia and Intensive Care Clinic | Marta Serwa |
| First Independent Teaching Hospital No. 1 | Lublin | II Department of Anesthesiology and Intensive Care | Miroslaw Czuczwar |
| Krakowski Szpital Specjalistyczny im. Jana Pawła II | Kraków | Thoracic Anaesthesia and Respiratory ICU | Mirosław Ziętkiewicz |
| Wroclaw Medical University | Wroclaw | Department of Anesthesiology and Intensve Therapy | Natalia Kozera |
| Szpital św.Anny W Miechowie | Miechów | Oddział Anestezjologii i Intensywnej Terapii | Paweł Nasiłowski |
| University Hospital in Krakow | Krakow | ICU Skawinska | Paweł Sendur |
| Infant Jesus Teaching Hospital | Warsaw | I Department of Anaesthesiology and Intensive Care | Paweł Zatorski |
| Regional Hospital in Bialystok | Bialystok | Department of Anaesthesiology and Intensive Care | Piotr Galkin |
| Opole University Hospital | Opole | Department of Anesthesiology and Intensive Care | Ryszard Gawda |
| University Hospital in Bialystok | Bialystok | Department of Anaesthesiology and Intensive Therapy | Urszula Kościuczuk |
| Dr Antoni Jurasz University Hospital in Bydgoszcz | Bydgoszcz | Department of Anesthesia and Critical Care | Waldemar Cyrankiewicz |
| Saint Lucas Hospital, Konskie | Konskie | Intensive Care Department | Wojciech Gola |

**Portugal**

| Centro Hospitalar do Porto | Porto | Serviço de Cuidados Intensivos 1 | Alexandre Fernandes Pinto |
| --- | --- | --- | --- |
| Hospital S. José, CHULC EPE | Lisboa | UCI Neurocríticos e Trauma | Ana Margarida Fernandes |
| Hospital São Francisco Xavier | Lisbon | Unidade Cuidados Intensivos Polivalente | Ana Rita Santos |
| Hospital da Luz | Lisboa | UCI Hospital da Luz | Cristina Sousa |
| Hospital de Viseu | Viseu | UCIP | Inês Barros |
| Hospital Professor Doutor Fernando Fonseca EPE | Amadora | Serviço de Medicina Intensiva SMI | Isabel Amorim Ferreira |
| Hospital Garcia de Orta - HGO | Almada | Serviço de Medicina Intensiva | Jacobo Bacariza Blanco |
| Hospital São Bernardo - CH Setúbal | Setúbal | Serviço de Cuidados Intensivos | João Teles Carvalho |
| Centro Hospitalar de Trás Montes e Alto Douro | Vila Real | Serviço de Medicina Intensiva | Jose Maia |
| Lusiadas Lisboa | Lisboa | UCI- Lusiadas | Nuno Candeias |
| CHMT-Abrantes | Abrantes | SMI | Nuno Catorze |

**Russia**

| Privolzhskiy District Medical Center | Nizhniy Novgorod | Department of Anesthesiology and Intensive Care | Vladislav Belskiy |
| --- | --- | --- | --- |

**Spain**

| Hospital De Bellvitge | Barcelona | UCI | Africa Lores |
| --- | --- | --- | --- |
| Hospital General Universitario de Albacete | Albacete | UCI Polivalente | Angela Prado Mira |
| Hospital Clinic of Barcelona | Barcelona | Respiratory Intensive Care Unit | Catia Cilloniz |
| Hospital Universitario Río Hortega | Valladolid | UVI Polivalente y Coronaria | David Perez-Torres |
| Universitario La Paz | Madrid | Surgical ICU | Emilio Maseda |
| General Universitario de Castellón | Castellón | Servicio de Medicina Intensiva | Enver Rodriguez |
| Hospital Universitario Río Hortega | Valladolid | UVI Neurocríticos Trauma y Quemados | Estefania Prol-Silva |
| Hospital de Tortosa Verge de la Cinta | Tortosa | Servei de Medicina Intensiva | Gaspar Masdeu Eixarch |
| Parc Taulí | Sabadell | Parc Taulí | Gemma Gomà |
| Clínico Universitario de Valencia | Valencia | Surgical Intensive Care Unit | Gerardo Aguilar |
| Hospital Universitario de Torrejon | Torrejon de Ardoz, Madrid | Intensive Care UNit | Gonzalo Navarro Velasco |
| Hospital General de Catalunya | Barcelona | HGC | Marián Irazábal Jaimes |
| Hospital Universitario Sagrado Corazon | Barcelona | Intensive Care Unit | Mercedes Ibarz Villamayor |
| Hospital reina Sofía | Murcia | Reina Sofía | Noemí Llamas Fernández |
| Complejo Hospitalario de Segovia | Segovia | ICU Segovia | Patricia Jimeno Cubero |
| Universitario de Getafe | Getafe | Intensive Care and Burn Unit | Sonia López-Cuenca |
| Germans Trias i Pujol Hospital | Badalona | General ICU | Teresa Tomasa |
| Centralsjukhuset i Karlstad | Karlstad | IVA | Anders Sjöqvist |

**Sweden**

| Umeå University | Umeå | Department of Surgical and Perioperative Sciences, Anestesiology and Intensive Care Medicine | Camilla Brorsson |
| --- | --- | --- | --- |
| Vrinnevisjukhuset | Norrköping | IVA Norrköping | Fredrik Schiöler |
| Sundsvall Hospital | Sundsvall | Sundsvall ICU | Henrik Westberg |
| Blekingesjukhuset | Karlskrona | Intensivvårdsavdelning 31 | Jessica Nauska |
| Alingsås Lasarett | Alingsås | Intensivvårdsavdelningen | Joakim Sivik |
| Västervikssjukhus | Västervik | IVA Västervikssjukhus | Johan Berkius |
| Sahlgrenska University Hospital/ Område 3/ Mölndals sjukhus | Göteborg | IVA avd 227 | Karin Kleiven Thiringer |
| Linköping University Hospital | Linköping | ICU Linköping | Lina De Geer |
| Linköping University Hospital | Linköping | Cardiothoracic Intensive Care Unit | Sten Walther |

**Switzerland**

| Hopitaux Universitaires de Genève | Geneva | Adult Intensive Care Unit | Filippo Boroli |
| --- | --- | --- | --- |
| University of Bern Inselspital | Bern | Department of Intensive Care Medicine | Joerg C. Schefold |
| Fribourg Hospital | Fribourg | Intensive Care Unit | Leila Hergafi |
| Centre Hospitalier Universitaire Vaudois | Lausanne | Service de médecine intensive adulte | Philippe Eckert |

**Turkey**

| Ordu University Training and Research Hospital | Ordu | General ICU | İsmail Yıldız |
| --- | --- | --- | --- |

**Ukraine**

| Dnipro Mechnikov Regional Clinical Hospital | Dnipro | Intensive Care Unit of Polytrauma | Ihor Yovenko |
| --- | --- | --- | --- |
| European Wellness Academy, Luhansk Regional Clinical Hospital | Luhansk | ICU 1 & 2 | Yuriy Nalapko |

**Wales**

| Glan Clwyd Hospital | Bodelwyddan | Critical Care | Richard Pugh |
| --- | --- | --- | --- |
